# Supplementary material for: Functional dual-slope frequency-domain near-infrared spectroscopy data interpreted with two- and three-layer models
Source: Biophotonics Discov. 2026 Mar 24;3(2):025003. doi: 10.1117/1.BIOS.3.2.025003 (PMC13107251; doi:10.1117/1.BIOS.3.2.025003)
Supplement: Supplementary file 1 [file BIOS_003_025003_SD001.pdf]

Supplementary Material: Functional dual-slope frequency-domain near-infrared spectroscopy data interpreted with two- and three-layer models

Jodee Frias,[*] Giles Blaney, Angelo Sassaroli, and Sergio Fantini
Tufts University, Department of Biomedical Engineering, Medford, MA, USA

1 Varying μ_s' of the Second Layer in the Three-Layer Medium

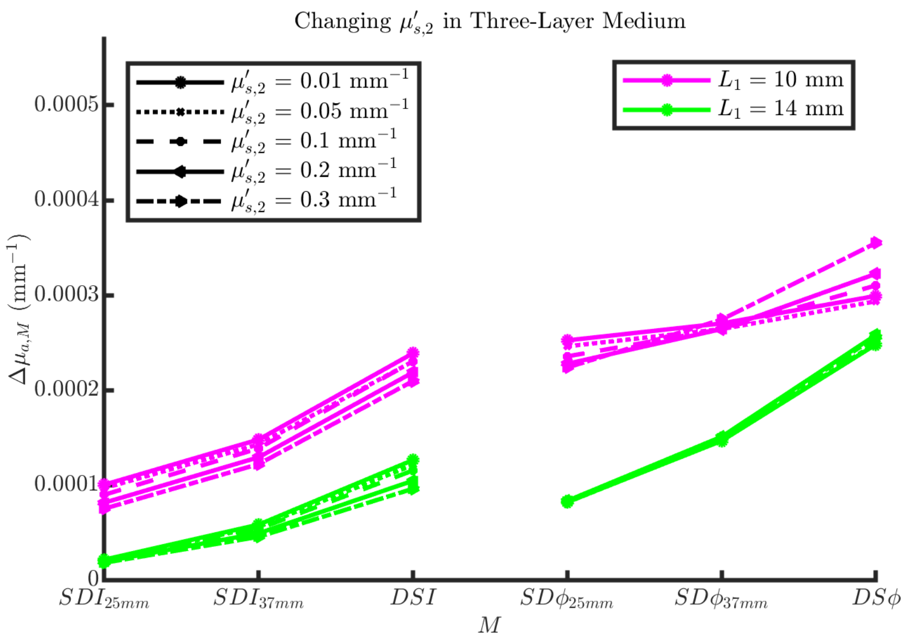

Figure S1. $\Delta\mu_{a,M}$ calculated from three-layer simulations, with L_1=10 mm (magenta) and L_1=14 mm (green). Properties held constant: $\Delta\mu_{a,3}$=0.001 mm^{-1}, $\mu_{a,1}$ = 0.017 mm^{-1}, $\mu_{a,2}$ = 0.0027 mm^{-1}, $\mu_{a,3}$ = 0.021 mm^{-1}, $\mu'_{s,1}$ = 0.75 mm^{-1}, $\mu'_{s,3}$ = 1.1 mm^{-1}. Varying properties: $\mu'_{s,2}$ is varied from 0.01 mm^{-1} to 0.3 mm^{-1}. While varying the $\mu'_{s,2}$ of the second layer representing the CSF-filled subarachnoid space, one can see that any of these explored $\mu'_{s,2}$ values allow for the *in vivo* hierarchical relationship to be satisfied.
